# GNSS/IMU/ODO/LiDAR-SLAM Integrated Navigation System Using IMU/ODO Pre-Integration

**DOI:** 10.3390/s20174702

**Published:** 2020-08-20

**Authors:** Le Chang, Xiaoji Niu, Tianyi Liu

**Affiliations:** GNSS Research Center, Wuhan University, 129 Luoyu Road, Wuhan 430079, China; changlesgg@whu.edu.cn (L.C.); liutianyi@whu.edu.cn (T.L.)

**Keywords:** GNSS, IMU, ODO, LiDAR-SLAM, pre-integration, graph optimization

## Abstract

In this paper, we proposed a multi-sensor integrated navigation system composed of GNSS (global navigation satellite system), IMU (inertial measurement unit), odometer (ODO), and LiDAR (light detection and ranging)-SLAM (simultaneous localization and mapping). The dead reckoning results were obtained using IMU/ODO in the front-end. The graph optimization was used to fuse the GNSS position, IMU/ODO pre-integration results, and the relative position and relative attitude from LiDAR-SLAM to obtain the final navigation results in the back-end. The odometer information is introduced in the pre-integration algorithm to mitigate the large drift rate of the IMU. The sliding window method was also adopted to avoid the increasing parameter numbers of the graph optimization. Land vehicle tests were conducted in both open-sky areas and tunnel cases. The tests showed that the proposed navigation system can effectually improve accuracy and robustness of navigation. During the navigation drift evaluation of the mimic two-minute GNSS outages, compared to the conventional GNSS/INS (inertial navigation system)/ODO integration, the root mean square (RMS) of the maximum position drift errors during outages in the proposed navigation system were reduced by 62.8%, 72.3%, and 52.1%, along the north, east, and height, respectively. Moreover, the yaw error was reduced by 62.1%. Furthermore, compared to the GNSS/IMU/LiDAR-SLAM integration navigation system, the assistance of the odometer and non-holonomic constraint reduced vertical error by 72.3%. The test in the real tunnel case shows that in weak environmental feature areas where the LiDAR-SLAM can barely work, the assistance of the odometer in the pre-integration is critical and can effectually reduce the positioning drift along the forward direction and maintain the SLAM in the short-term. Therefore, the proposed GNSS/IMU/ODO/LiDAR-SLAM integrated navigation system can effectually fuse the information from multiple sources to maintain the SLAM process and significantly mitigate navigation error, especially in harsh areas where the GNSS signal is severely degraded and environmental features are insufficient for LiDAR-SLAM.

## 1. Introduction

Navigation systems that integrate multiple sensors and provide information for high data rates, high accuracy, and all-weather capability will become the core components of platforms such as autonomous driving and intelligent robots in the near future. At present, the GNSS (global navigation satellite system)/INS (integrated navigation system) mainly based on inertial navigation and supplemented by satellite navigation is most widely used. In the GNSS/INS integrated navigation system, GNSS/INS information have distinctively complementary characteristics and are fused by the Kalman filter [1] to improve navigation performance. However, GNSS/INS navigation system accuracy depends on good GNSS signals, especially the system using low-cost MEMS (micro-electro mechanical system)-IMU (inertial measurement unit).

**Figure 1:**
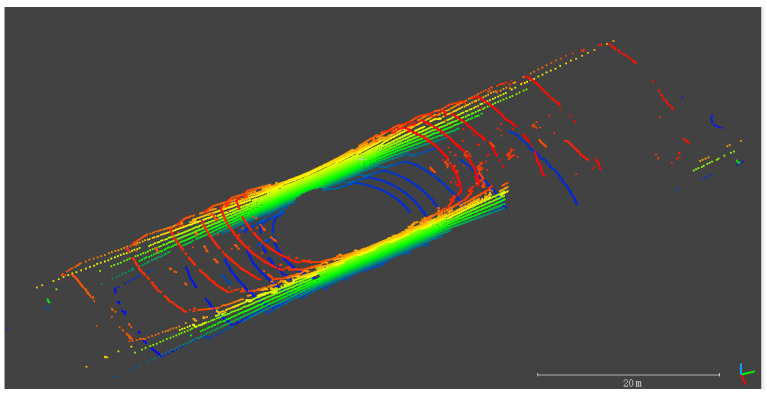
Point clouds in the tunnel colored by the height.

In recent years, with the improvement of computer performance, SLAM (simultaneous localization and mapping) technology that uses remote sensors for navigation has been widely used in vehicle navigation. SLAM mainly obtains the pose by matching the observed environmental features with the feature map while moving and simultaneously updates the feature map to achieve autonomous positioning [2]. SLAM generally uses cameras or LiDAR (light detection and ranging) as sensors. Both sensors have their strengths and weaknesses. Compared with cameras, LiDAR can directly obtain 3D structural information in the environment, which is less affected by light and weather. Yet, it still has shortcomings, such as higher cost and lower resolution. LiDAR-SLAM [3,4,5,6,7,8,9,10] uses environmental features extracted from the point cloud to match and obtain pose changes for navigation. Similar to INS, LiDAR-SLAM is also a type of recursive navigation and its navigation error gradually diverges with the moving distance. The closed-loop correction is usually used to reduce such recursion errors. However, in the outdoor environment, closed-loop conditions are difficult to meet. Therefore, the fusion of the GNSS/INS integrated navigation system and SLAM can help reduce SLAM dependence on closed-loop correction and improve navigation performance when GNSS cannot work. Therefore, this fulfills the complementary advantages of multiple sensors. There are two main methods for multi-sensor data fusion: filtering [11,12,13,14] and graph optimization [15,16,17]. Compared with the former, the latter is more accurate and robust, but time-consuming [18]. In our previous work [19], we optimized IMU (inertial measurement unit) information fusion based on a cartographer [16]. However, the matching effect by the 3D probabilistic map matching [19] is not good in elevation, roll, and pitch for the LiDAR, such as VLP-16 whose vertical resolution is low. Moreover, in areas where environmental features are limited and GNSS are not available, such as is the tunnel case in Figure 1, the GNSS/IMU/LiDAR-SLAM integrated navigation system cannot work well.

For the ground wheel carrier such as cars and wheel robots, auxiliary methods such as odometer (ODO) and non-holonomic constraint (NHC) can usually be added in the GNSS/INS integrated navigation system to suppress the divergence of navigation errors when the GNSS signal is interrupted [20,21,22]. For land vehicles, the odometer is an economical and conveniently installed sensor [23]. NHC is a virtual velocity observation constructed by ignoring the movement of land vehicles in vertical and lateral directions, and does not rely on any sensors [21,22]. Similarly, such kind of vehicle aiding information can be added into the GNSS/IMU/LiDAR-SLAM system for a more comprehensive navigation performance. Therefore, in view of the low vertical resolution of VLP-16 and the possible failure of LiDAR-SLAM, this paper implemented a GNSS/IMU/ODO/LiDAR-SLAM integrated navigation system based on the LeGO-LOAM (Lightweight and ground-optimized lidar odometry and mapping) [5] feature matching method and the solution [19] for road environments. Odometer information is usually converted to the forward speed for assistance [24,25]. Although this method is convenient, it is difficult to obtain the accurate speed of the auxiliary moment because the original measurement information of the odometer is mileage. Considering the existing IMU pre-integration in the graph optimization algorithm [19], this paper converted odometer mileage into displacement through pre-integration, forming the IMU/odometer (ODO) pre-integration constraint to replace IMU pre-integration.

**Figure 2:**
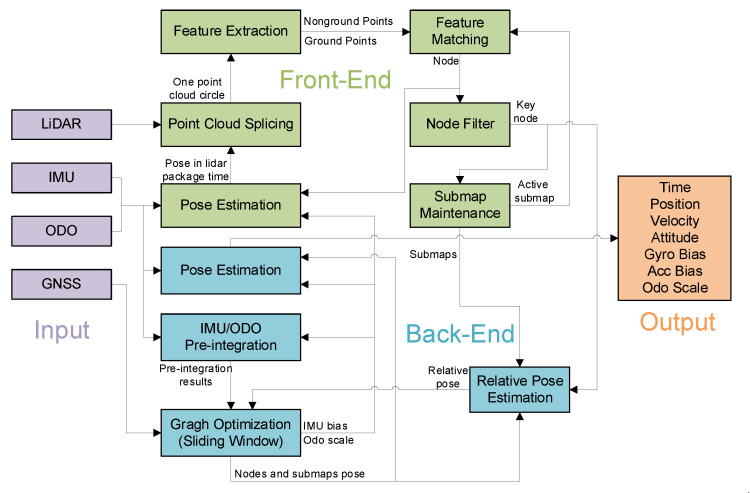
System overview of the GNSS (global navigation satellite system)/IMU (inertial measurement unit)/odometer (ODO)/LiDAR (light detection and ranging)-SLAM (simultaneous localization and mapping) integrated navigation system.

An overview of the proposed GNSS/IMU/ODO/LiDAR-SLAM integrated navigation system is shown in Figure 2. This system is mainly composed of the front- and back-end. The front-end is used for point cloud matching. Firstly, the dead reckoning results were obtained using IMU/ODO and the point cloud data packets obtained through LiDAR were motion-compensated with the dead reckoning results and stitched into a circle of point clouds. Then, the ground feature points and non-ground feature points extracted from the point clouds were used for feature matching to obtain the pose of the vehicle. Further, the feature points and pose were noted as a node. Finally, nodes that met conditions of motion filtering were added into the feature submaps. The back-end used graph optimization to fuse information from multiple sensors. There are three cost functions for nonlinear optimization: GNSS positioning results, IMU/ODO pre-integration results, and the relative pose between all key nodes and related submaps. Because of the heavy workload of traversing to find related submaps, the branch and bound method [10] was adopted to speed up the search. In order to prevent the amount of calculation from increasing over time, a sliding window was applied to keep the number of optimized variables relatively stable. The final navigation results were obtained using the MEMS-IMU mechanization starting from the latest node pose in the sliding window.

## 2. Materials and Methods

### 2.1. Coordinate System

**Figure 3:**
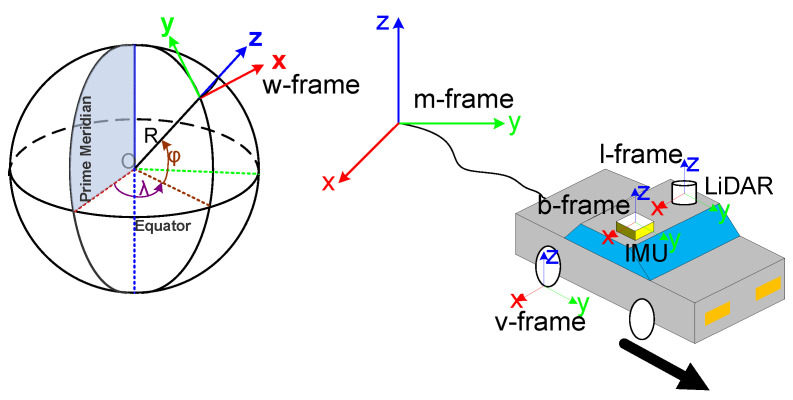
Coordinate systems.

The proposed system has four different sensors, so it needs fuse the information in different coordinate systems (as shown in Figure 3).

(1) b-frame: The coordinate system of the IMU with the IMU center as the origin, the X-axis pointing right, the Y-axis pointing forwards and the Z-axis pointing up.
(2) l-frame: The coordinate system of the LiDAR with the LiDAR center as the origin, the X-axis pointing right, the Y-axis pointing forwards, and the Z-axis pointing up.
(3) v-frame: The coordinate system of the vehicle with the tangent point of the wheel where the odometer installed to the ground as the origin, the X axis pointing right, the Y axis pointing forwards, and the Z-axis pointing up.
(4) w-frame: The coordinate system of the GNSS positioning results with the initial GNSS position as the origin, the X-axis pointing east, the Y-axis pointing north, and the Z-axis pointing up.
(5) m-frame: The coordinate system of LiDAR-SLAM with the initial SLAM position as the origin and the coordinate axis coinciding with the b-frame on initialization.

### 2.2. Front-End

#### 2.2.1. Pose Estimation

According to Chang [19], the MEMS-IMU mechanization can be used to update the position, velocity, and attitude:

(1) $$\begin{array}{l} \Delta t_{i}=t_{i}-t_{i-1}\\ v_{mb_{i}}^{m}=v_{mb_{i-1}}^{m}+C_{b_{i-1}}^{m}v_{b_{i-1}b_{i}}^{b_{i-1}}+g^{m}\Delta t_{i}\\ P_{mb_{i}}^{m}=P_{mb_{i-1}}^{m}+v_{mb_{i-1}}^{m}\Delta t_{i}+\frac{1}{2}g^{m}\Delta t_{i}^{2}+\frac{1}{2}C_{b_{i-1}}^{m}v_{b_{i-1}b_{i}}^{b_{i-1}}\Delta t_{i}\\ q_{b_{i}}^{m}=q_{b_{i-1}}^{m}⊗q_{b_{i}}^{b_{i-1}} \end{array}$$

where *b_i_* is the b-frame at *t_i_*; **g**^*m*^ is gravity in m-frame; $v_{mb}^{m}$ is the velocity of b-frame relative to m-frame projected on the m-frame; $P_{mb}^{m}$ is the translation of b-frame relative to m-frame projected on the m-frame; $q_{b}^{m}$ is the quaternion; $C_{b_{i-1}}^{m}$ is the direction cosine matrix of $q_{b}^{m}$; $v_{b_{i-1}b_{i}}^{b_{i-1}}$ and $q_{b_{i}}^{b_{i-1}}$ are the increments in velocity and attitude from *t*_*i*−1_ to *t_i_*, respectively:

(2) $$\begin{array}{c} v_{b_{i-1}b_{i}}^{b_{i-1}}\approx \Delta v_{f,t_{i}}^{b}+\frac{1}{2}\Delta \theta_{t_{i}}\times \Delta v_{f,t_{i}}^{b}\\ q_{b_{i}}^{b_{i-1}}\approx \left[\begin{array}{c} \cos\left\|0.5\Delta \theta_{t_{i}}\right\|\\ \frac{\sin\left\|0.5\Delta \theta_{t_{i}}\right\|}{\left\|\Delta \theta_{t_{i}}\right\|}\Delta \theta_{t_{i}} \end{array}\right]\\ \Delta v_{f,t_{i}}^{b}=\int_{t_{i-1}}^{t_{i}}f^{b_{t}}dt=\int_{t_{i-1}}^{t_{i}}\left[{\tilde{f}}^{b_{t}}-b_{a}\left(t\right)\right]dt\\ \Delta \theta_{t_{i}}=\int_{t_{i-1}}^{t_{i}}\omega^{b_{t}}dt=\int_{t_{i-1}}^{t_{i}}\left[{\tilde{\omega }}^{b_{t}}-b_{g}\left(t\right)\right]dt \end{array}$$

where ${\tilde{f}}^{b_{t}}$ and ${\tilde{\omega }}^{b_{t}}$ are the specific force and the angular rate measured by the IMU, respectively; **b***_a_* and **b***_g_* are the biases of the accelerometer and gyroscope, respectively.

When the system has the odometer observation data, pose estimation can be obtained from the synchronously collected gyroscope output of the IMU and odometer output. In the estimation process, it is generally assumed that the vertical and lateral speed of the vehicle in v-frame is zero, which is called NHC. In another word, the vertical and lateral movements of the contact point between the wheel with the odometer and the ground are ignored. Therefore, the odometer increment $s_{v_{i}}^{v_{i-1}}$ can be expressed as Equation (3):

(3) $$s_{v_{i}}^{v_{i-1}}={\left[\begin{array}{ccc} 0 & {\tilde{s}}^{v_{i}}\left(1+s_{odo}\right) & 0 \end{array}\right]}^{T}$$

where ${\tilde{s}}^{v_{i}}$ is the odometer output from *t_i_*_−1_ to *t_i_* and *S_odo_* is the odometer scale factor.

The pose estimation of the IMU in m-frame requires the odometry increment $s_{b_{i}}^{b_{i-1}}$ in b-frame:

(4) $$s_{b_{i}}^{b_{i-1}}=\int_{t_{i-1}}^{t_{i}}v_{b_{i-1}b_{t}}^{b_{i-1}}dt$$

The velocity conversion between the b-frame and v-frame is expressed as Equation (5):

(5) $$v_{mv_{t}}^{m}=v_{mb_{t}}^{m}+C_{b_{t}}^{m}\left[\omega_{mb_{t}}^{b_{t}}\times \right]I_{odo}^{b}$$

where $I_{odo}^{b}$ is the lever arm of the odometry.

Substituting Equation (5) into Equation (6):

(6) $$\begin{array}{cc} s_{b_{i}}^{b_{i-1}} & =\int_{t_{i-1}}^{t_{i}}C_{m}^{b_{i-1}}v_{mb_{t}}^{m}dt\\ & =\int_{t_{i-1}}^{t_{i}}\left(v_{b_{i-1}v_{t}}^{b_{i-1}}-C_{b_{t}}^{b_{i-1}}\left[\omega_{mb_{t}}^{b_{t}}\times \right]I_{odo}^{b}\right)dt\\ & =\int_{t_{i-1}}^{t_{i}}\left(C_{b_{t}}^{b_{i-1}}C_{v}^{b}v_{b_{i-1}v_{t}}^{v_{t}}\right)dt-\int_{t_{i-1}}^{t_{i}}\left(C_{b_{t}}^{b_{i-1}}\left[\omega_{mb_{t}}^{b_{t}}\times \right]I_{odo}^{b}\right)dt \end{array}$$

where $C_{v}^{b}$ is the IMU mounting angles, which can be obtained by the alignment calibration [26].

The odometer sampling interval (*t_i_*_−1_~*t_i_*) is small, thus the attitude can be considered unchanged:

(7) $$\begin{array}{cc} \int_{t_{i-1}}^{t_{i}}\left(C_{b_{t}}^{b_{i-1}}C_{v}^{b}v_{b_{i-1}v_{t}}^{v_{t}}\right)dt & \approx \int_{t_{i-1}}^{t_{i}}\left(C_{b_{i-1}}^{b_{i-1}}C_{v}^{b}v_{v_{i-1}v_{t}}^{v_{i-1}}\right)dt\\ & \approx C_{v}^{b}s_{v_{i}}^{v_{i-1}} \end{array}$$

The m-frame and the *b*_*i*−1_-frame are relatively fixed during the integration process:

(8) $$\int_{t_{i-1}}^{t_{i}}\left(C_{b_{t}}^{b_{i-1}}\left[\omega_{mb_{t}}^{b_{t}}\times \right]I_{odo}^{b}\right)dt\approx \int_{t_{i-1}}^{t_{i}}\left(C_{b_{t}}^{b_{i-1}}\left[\omega_{b_{i-1}b_{t}}^{b_{t}}\times \right]I_{odo}^{b}\right)dt$$

According to the differential equation of the direction cosine matrix, we have Equation (9):

(9) $$\begin{array}{cc} \int_{t_{i-1}}^{t_{i}}\left(C_{b_{t}}^{b_{i-1}}\left[\omega_{b_{i-1}b_{t}}^{b_{t}}\times \right]I_{odo}^{b}\right)dt & =\int_{t_{i-1}}^{t_{i}}\left({\dot{C}}_{b_{t}}^{b_{i-1}}I_{odo}^{b}\right)dt\\ & =\left(C_{b_{i}}^{b_{i-1}}-C_{b_{i-1}}^{b_{i-1}}\right)I_{odo}^{b}\\ & =C_{b_{i}}^{b_{i-1}}I_{odo}^{b}-I_{odo}^{b} \end{array}$$

In summary, Formula Equation (6) can be simplified as:

(10) $$s_{b_{i}}^{b_{i-1}}\approx C_{v}^{b}s_{v_{i}}^{v_{i-1}}-C_{b_{i}}^{b_{i-1}}I_{odo}^{b}+I_{odo}^{b}$$

Therefore, the position and attitude can be updated by the gyro output of the IMU and odometer output as follows:

(11) $$\begin{array}{c} P_{mb_{i}}^{m}=P_{mb_{i-1}}^{m}+C_{b_{i-1}}^{m}\left(C_{v}^{b}s_{v_{i}}^{v_{i-1}}-C_{b_{i}}^{b_{i-1}}I_{odo}^{b}+I_{odo}^{b}\right)\\ q_{b_{i}}^{m}=q_{b_{i-1}}^{m}⊗q_{b_{i}}^{b_{i-1}} \end{array}$$

#### 2.2.2. Feature Extraction

**Figure 4:**
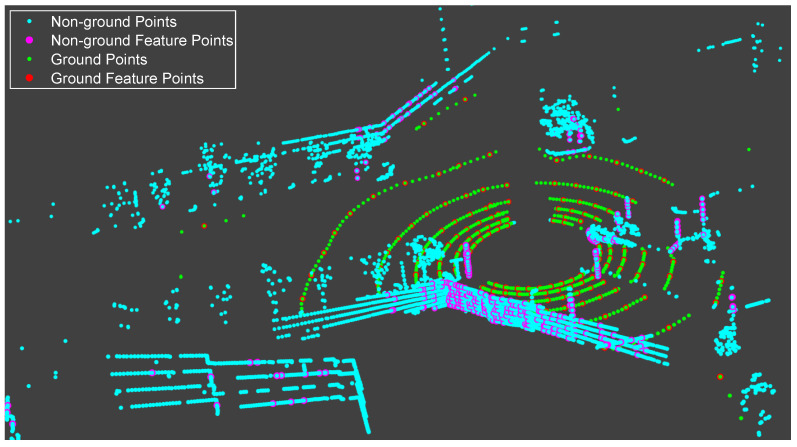
The feature points in typical road environment.

After obtaining a circle of point clouds with motion compensation, noted as one node [19], it follows the matching method of a cartographer, using the voxel filtered points as feature points to match with the 3D probability map. Because of the relatively large matching errors in elevation, roll, and pitch, this method is not suitable for VLP-16 whose vertical resolution is low. The low vertical resolution makes the point clouds in the vertical direction and the ground not dense enough, and the 3D probability map matching cannot estimate the elevation, roll, and pitch. Therefore, referring to the feature matching method of LeGO-LOAM, this paper extracts feature points and finally gets ground points, ground feature points, non-ground points, and non-ground feature points, as shown in Figure 4. The ground feature points are used to estimate elevation, roll, and pitch. The non-ground feature points are used to estimate the horizontal position and yaw. The specific feature extraction methods are not repeated here.

#### 2.2.3. Submap Maintenance

Similar to a cartographer [16], this paper also uses submaps to manage feature maps; two active submaps are maintained. The first created submap is used for feature matching and the feature points after the feature matching are transformed into the coordinates of the two submaps to update the two submaps, respectively. When the accumulated mileage of the submap created first reached 100 m, the submap was fixed and a new submap was created. In this cycle, two submaps were always active and there was an overlap area between adjacent submaps.

Considering that the feature point-based matching method is inconvenient for the closed-loop and the feature objects (such as building surfaces, trees, street lights, etc.) in urban environments generally do not change much in the vertical direction, point-to-surface distance matching for ground feature points and 2D probability map matching [10] for non-ground feature points were applied in this paper. Therefore, each submap contained a K-D tree formed by ground points and a 2D probability map formed by non-ground points. When the submap was updated, non-ground points were used to update the 2D probability map, and ground points were used for the K-D tree update.

#### 2.2.4. Feature Matching

The optimized pose can be obtained using the pose in Section 2.2.1. to optimize elevation, roll, and pitch with the ground point matching (Equation (12)). Then, we used the obtained result to optimize the horizontal position and yaw with the non-ground feature points, as in Equation (13). Moreover, the nonlinear optimization solver used in this paper was Ceres [27].

(1) Ground Point Match
  (12) $$\underset{q_{l}^{m},P_{ml}^{m}}{\arg\min}{\sum_{k=1}^{K}\left(n_{k}\cdot \left(C_{l}^{m}p_{k}^{l}+P_{ml}^{m}\right)+d_{k}\right)}^{2}$$
  where *K* is the number of ground feature points contained in the node; $p_{k}^{l}$ is the k-th point’s position in l-frame; **n***_k_*, *d_k_* are the plane normal and plane distance obtained by fitting all points with a radius of 0.5 m around the *k*-th ground feature point.
(2) Probability Map Match
  (13) $$\underset{q_{l}^{m},P_{ml}^{m}}{\arg\min}{\sum_{k=1}^{K}\left(1-Map\left(C_{l}^{m}p_{k}^{l}+P_{ml}^{m}\right)\right)}^{2}$$
  where *K* is the number of non-ground feature points contained in the node; *Map* is the mapping function from the coordinates of the point in the 2D probability map to the probability value [10].

#### 2.3. Back-End

The information fusion method used in this paper was graph optimization and the optimization parameter is set to **χ**, as shown in Equation (14):

(14) $$\begin{array}{c} \chi =\left[x_{1},x_{2}\cdots x_{N},y_{1},y_{2}\cdots y_{M},P_{wm}^{w},q_{m}^{w}\right]\\ x_{k}=\left[P_{mb_{k}}^{m},v_{mb_{k}}^{m},q_{b_{k}}^{m},b_{a}\left(t_{k}\right),b_{g}\left(t_{k}\right),s_{odo}\left(t_{k}\right)\right],k\in \left[1,N\right]\\ y_{k}=\left[P_{ms_{k}}^{m},q_{s_{k}}^{m}\right],k\in \left[1,M\right] \end{array}$$

where **x***_k_* is composed of the position, velocity, and attitude of the IMU in m-frame, the accelerometer bias, the gyroscope bias, and the odometer scale factor at *t_k_*; *N* is the number of nodes; **y***_k_* is the position and attitude of the submap in m-frame at *t_k_*; and *M* is the number of submaps.

The cost functions are found in Equation (15):

(15) $$\underset{\chi }{\arg\min}\left\{\begin{array}{c} \sum_{i\in \alpha }E_{GNSS}^{2}\left(x_{i},P_{wm}^{w},q_{m}^{w},p_{g_{i}}^{w},l_{g}^{b},\sigma_{p}\right)+\sum_{i\in \beta ,j\in \kappa }E_{LiDAR}^{2}\left(x_{i},y_{j},P_{bl}^{b},q_{l}^{b},P_{s_{j}l_{i}}^{s_{j}},q_{l_{i}}^{s_{j}},\sigma_{ij}\right)\\ +\sum_{i=1}^{N-1}E_{IMU/ODO}^{2}\left(x_{i},x_{i+1},z_{i+1}^{i},\sigma_{z}\right) \end{array}\right\}$$

where $E_{GNSS}^{2}$, $E_{LiDAR}^{2}$, and $E_{IMU/ODO}^{2}$ are the cost functions of GNSS, LiDAR, and IMU/ODO, respectively; $p_{g}^{w}$ is the position in w-frame converted from the geodetic coordinate obtained by the GNSS receiver [28]; $l_{g}^{b}$ is the GNSS antenna’s lever arm; **σ***_p_* is the standard deviation of $p_{g}^{w}$; *α* is the nodes set with the GNSS positioning result; $P_{bl}^{b}$ and $q_{l}^{b}$ are the extrinsic calibration parameters of the LiDAR [29]; $P_{s_{j}l_{i}}^{s_{j}},q_{l_{i}}^{s_{j}}$ are the relative position and attitude of the node *l_i_* and the submap *s_j_*; **σ***_ij_* is the standard deviation of $P_{s_{j}l_{i}}^{s_{j}},q_{l_{i}}^{s_{j}}$; *β* is the nodes set; and κ is the submaps set; $z_{i+1}^{i}$ is the pre-integration result between the *t_i_* node and the *t*_*i*+1_ node; **σ**_z_ is the standard deviation of **z**.

According to Chang [19], the cost functions of GNSS and LiDAR can be obtained:

(16) $$\begin{array}{cc} E_{GNSS}^{2}\left(x_{i},P_{wm}^{w},q_{m}^{w},p_{g_{i}}^{w},l_{g}^{b},\sigma_{p}\right) & =e{\left(x_{i},P_{wm}^{w},q_{m}^{w},P_{g_{i}}^{w},l_{g}^{b}\right)}^{T}{\left(\sigma_{p}^{2}\right)}^{-1}e\left(x_{i},P_{wm}^{w},q_{m}^{w},p_{g_{i}}^{w},l_{g}^{b}\right)\\ e\left(x_{i},P_{wm}^{w},q_{m}^{w},p_{g_{i}}^{w},l_{g}^{b}\right) & =C_{m}^{w}\left(C_{b_{i}}^{m}l_{g}^{b}+P_{mb_{i}}^{m}\right)+P_{wm}^{w}-p_{g_{i}}^{w} \end{array}$$

(17) $$\begin{array}{cc} E_{LiDAR}^{2}\left(x_{i},y_{j},P_{bl}^{b},q_{l}^{b},P_{s_{j}l_{i}}^{s_{j}},q_{l_{i}}^{s_{j}},\sigma_{ij}\right) & =e{\left(x_{i},y_{j},P_{bl}^{b},q_{l}^{b},P_{s_{j}l_{i}}^{s_{j}},q_{l_{i}}^{s_{j}}\right)}^{T}{\left(\sigma_{ij}^{2}\right)}^{-1}e\left(x_{i},y_{j},P_{bl}^{b},q_{l}^{b},P_{s_{j}l_{i}}^{s_{j}},q_{l_{i}}^{s_{j}}\right)\\ e\left(x_{i},y_{j},P_{bl}^{b},q_{l}^{b},P_{s_{j}l_{i}}^{s_{j}},q_{l_{i}}^{s_{j}}\right) & =\left[\begin{array}{c} {\left(C_{s_{j}}^{m}\right)}^{-1}\left[p_{mb_{i}}^{m}+C_{b_{i}}^{m}p_{bl}^{b}-p_{ms_{j}}^{m}\right]-p_{s_{j}l_{i}}^{s_{j}}\\ {\left[{\left(q_{b_{i}}^{m}⊗q_{l}^{b}\right)}^{-1}⊗q_{s_{j}}^{m}⊗q_{l_{i}}^{s_{j}}\right]}_{xyz} \end{array}\right] \end{array}$$

where *e* is the residual function and **[q]**_xyz_ is the equivalent rotation vector of **q**.

According to Equation (11) and the IMU pre-integration formula in VINS (Visual-Inertial Navigation System) [30], the IMU/ODO pre-integration formula can be obtained:

(18) $$\begin{array}{c} \alpha_{b_{k-1}}^{b_{k-1}}=0,\beta_{b_{k-1}}^{b_{k-1}}=0,s_{b_{k-1}}^{b_{k-1}}=0,q_{b_{k-1}}^{b_{k-1}}=I\\ \beta_{b_{i}}^{b_{k-1}}=\beta_{b_{i-1}}^{b_{k-1}}+C_{b_{i-1}}^{b_{k-1}}v_{b_{i-1}b_{i}}^{b_{i-1}}\\ \alpha_{b_{i}}^{b_{k-1}}=\alpha_{b_{i-1}}^{b_{k-1}}+\frac{1}{2}C_{b_{i-1}}^{b_{k-1}}v_{b_{i-1}b_{i}}^{b_{i-1}}\Delta t_{i}\\ s_{b_{i}}^{b_{k-1}}=s_{b_{i-1}}^{b_{k-1}}+C_{b_{i-1}}^{b_{k-1}}\left(C_{v}^{b}s_{v_{i}}^{v_{i-1}}-C_{b_{i}}^{b_{i-1}}I_{odo}^{b}+I_{odo}^{b}\right)\\ q_{b_{i}}^{b_{k-1}}=q_{b_{i-1}}^{b_{k-1}}⊗q_{b_{i}}^{b_{i-1}} \end{array}$$

where $\alpha_{b_{i}}^{b_{k-1}}$, $\beta_{b_{i}}^{b_{k-1}}$, $q_{b_{i}}^{b_{k-1}}$, and $s_{b_{i}}^{b_{k-1}}$ are the pre-integration form of the position increment, velocity increment, attitude increment, and odometer increment. These increments are all only related to the original output, IMU bias, and odometer scale, and are independent of the position and attitude at the starting point of the integration. *t_i_* is the sampling moment of the IMU and the odometer between *t*_*k*−1_ and *t_k_*, *t_i_* ∈ [*t*_*k*−1_, *t_k_*]. The IMU bias and the odometer scale factor at *t*_*k*−1_ are used to correct the original outputs of the IMU and the odometer between *t*_*k*−1_ and *t_k_*, and the IMU bias and the odometer scale factor between the two adjacent nodes are considered unchanged.

The differential equation of the odometer pre-integration $s_{b_{t}}^{b_{k-1}}$ can be derived from Formula Equation (5):

(19) $$\begin{array}{cc} {\dot{s}}_{b_{t}}^{b_{k-1}} & =v_{b_{k-1}b_{t}}^{b_{k-1}}\\ & =v_{b_{k-1}v_{t}}^{b_{k-1}}-C_{b_{t}}^{b_{k-1}}\left[\omega_{b_{k-1}b_{t}}^{b_{t}}\times \right]I_{odo}^{b}\\ & =C_{b_{t}}^{b_{k-1}}C_{v}^{b}v_{b_{k-1}v_{t}}^{v_{t}}\left(1+s_{odo}\right)-C_{b_{t}}^{b_{k-1}}\left[\omega^{b_{t}}\times \right]I_{odo}^{b} \end{array}$$

Perturbations on both sides can be written as Equation (20):

(20) $$\begin{array}{cc} {\dot{s}}_{b_{t}}^{b_{k-1}}+\delta {\dot{s}}_{b_{t}}^{b_{k-1}} & =C_{b_{t}}^{b_{k-1}}\left(I+\left[\delta \theta_{b_{t}}^{b_{k-1}}\times \right]\right)(C_{v}^{b}\left(v_{b_{k-1}v_{t}}^{v_{t}}+\delta v_{odo}\right)\left(1+s_{odo}+\delta s_{odo}\right)\\ & -\left[\left(\omega^{b_{t}}+\delta \omega^{b}\right)\times \right]I_{odo}^{b}) \end{array}$$

where $\delta \theta_{b_{t}}^{b_{k-1}}$ is the error of $C_{b_{t}}^{b_{k-1}}$ in the form of equivalent rotation vector; *δ***v***_odo_* is the error of the odometer velocity; *δS_odo_* is the error of the odometer scale factor; *δ***ω**^b^ is the error of the gyroscope observation.

Ignoring the second-order small quantization and after simplifying, Equation (20) can be written as Equation (21):

(21) $$\begin{array}{cc} \delta {\dot{s}}_{b_{t}}^{b_{k-1}} & \approx C_{b_{t}}^{b_{k-1}}C_{v}^{b}\left(1+s_{odo}\right)\delta v_{odo}\\ & +C_{b_{t}}^{b_{k-1}}\left[I_{odo}^{b}\times \right]\delta \omega^{b}+C_{b_{t}}^{b_{k-1}}C_{v}^{b}v_{b_{k-1}v_{t}}^{v_{t}}\delta s_{odo}\\ & -C_{b_{t}}^{b_{k-1}}\left[\left(C_{v}^{b}v_{b_{k-1}v_{t}}^{v_{t}}\left(1+s_{odo}\right)-\omega^{b_{t}}\times I_{odo}^{b}\right)\times \right]\delta \theta_{b_{t}}^{b_{k-1}} \end{array}$$

Combining the derivation of IMU pre-integration in Chang [19] and modeling the odometry scale factor as a random walk [31], the differential equation of IMU/ODO pre-integration can be obtained:

(22) $$\delta {\dot{z}}_{t}^{t_{k-1}}=F\left(t\right)\delta z_{t}^{t_{k-1}}+G\left(t\right)w\left(t\right)$$

where

$$\begin{array}{c} F\left(t\right)=\left[\begin{array}{ccccccc} 0_{3\times 3} & I_{3\times 3} & 0_{3\times 3} & 0_{3\times 3} & 0_{3\times 3} & 0_{3\times 3} & 0_{3\times 1}\\ 0_{3\times 3} & 0_{3\times 3} & -C_{b_{t}}^{b_{k-1}}\left[f^{b_{t}}\times \right] & -C_{b_{t}}^{b_{k-1}} & 0_{3\times 3} & 0_{3\times 3} & 0_{3\times 1}\\ 0_{3\times 3} & 0_{3\times 3} & -\left[\omega^{b_{t}}\times \right] & 0_{3\times 3} & -I_{3\times 3} & 0_{3\times 3} & 0_{3\times 1}\\ 0_{3\times 3} & 0_{3\times 3} & 0_{3\times 3} & -1/\tau_{a} & 0 & 0_{3\times 3} & 0_{3\times 1}\\ 0_{3\times 3} & 0_{3\times 3} & 0_{3\times 3} & 0_{3\times 3} & -1/\tau_{g} & 0_{3\times 3} & 0_{3\times 1}\\ 0_{3\times 3} & 0_{3\times 3} & -C_{b_{t}}^{b_{k-1}}\left[\left(\begin{array}{l} C_{v}^{b}v_{b_{k-1}v_{t}}^{v_{t}}\left(1+s_{odo}\right)\\ -\omega^{b_{t}}\times I_{odo}^{b} \end{array}\right)\times \right] & 0_{3\times 3} & C_{b_{t}}^{b_{k-1}}\left[I_{odo}^{b}\times \right] & 0_{3\times 3} & C_{b_{t}}^{b_{k-1}}C_{v}^{b}v_{b_{k-1}v_{t}}^{v_{t}}\\ 0_{1\times 3} & 0_{1\times 3} & 0_{1\times 3} & 0_{1\times 3} & 0_{1\times 3} & 0_{1\times 3} & 0 \end{array}\right]\\ \delta z_{t}^{t_{k-1}}={\left[\begin{array}{ccccccc} \delta \alpha_{b_{t}}^{b_{k-1}} & \delta \beta_{b_{t}}^{b_{k-1}} & \delta \theta_{b_{t}}^{b_{k-1}} & \delta b_{a} & \delta b_{g} & \delta s_{b_{t}}^{b_{k-1}} & \delta s_{odo} \end{array}\right]}^{T}\\ G\left(t\right)=\left[\begin{array}{cccccc} 0_{3\times 3} & 0_{3\times 3} & 0_{3\times 3} & 0_{3\times 3} & 0_{3\times 3} & 0_{3\times 1}\\ C_{b_{t}}^{b_{k-1}} & 0_{3\times 3} & 0_{3\times 3} & 0_{3\times 3} & 0_{3\times 3} & 0_{3\times 1}\\ 0_{3\times 3} & I_{3\times 3} & 0_{3\times 3} & 0_{3\times 3} & 0_{3\times 3} & 0_{3\times 1}\\ 0_{3\times 3} & 0_{3\times 3} & I_{3\times 3} & 0_{3\times 3} & 0_{3\times 3} & 0_{3\times 1}\\ 0_{3\times 3} & 0_{3\times 3} & 0_{3\times 3} & I_{3\times 3} & 0_{3\times 3} & 0_{3\times 1}\\ 0_{3\times 3} & C_{b_{t}}^{b_{k-1}}\left[I_{odo}^{b}\times \right] & 0_{3\times 3} & 0_{3\times 3} & C_{b_{t}}^{b_{k-1}}C_{v}^{b}\left(1+s_{odo}\right) & 0_{3\times 1}\\ 0_{1\times 3} & 0_{1\times 3} & 0_{1\times 3} & 0_{1\times 3} & 0_{1\times 3} & 1 \end{array}\right]\\ w\left(t\right)={\left[\begin{array}{cccccc} w_{a} & w_{g} & w_{ca} & w_{cg} & w_{odo} & w_{s} \end{array}\right]}^{T} \end{array}$$

where **w***_a_* is the white noises of the accelerometer observations; **w***_g_* is the white noises of the gyroscope observations; *τ_a_*, *τ_g_* are the correlation time of the first-order Gauss-Markov process; **w***_ca_*, **w***_cg_* are the white noise of the first-order Gauss-Markov process; **w**_odo_ is the white noises of the odometry observations; *w_s_* is the white noise of the random walk process.

Finally, the IMU/ODO pre-integration cost function is obtained with Equation (23):

(23) $$\begin{array}{l} E_{IMU/ODO}^{2}\left(x_{k-1},x_{k},z_{t_{k}}^{t_{k-1}},\sigma_{z}\right)=e{\left(x_{k-1},x_{k},z_{t_{k}}^{t_{k-1}}\right)}^{T}{\left(\sigma_{z}^{2}\right)}^{-1}e\left(x_{k-1},x_{k},z_{t_{k}}^{t_{k-1}}\right)\\ e\left(x_{k-1},x_{k},z_{t_{k}}^{t_{k-1}}\right)=\left[\begin{array}{c} C_{m}^{b_{k-1}}\left(p_{mb_{k}}^{m}-p_{mb_{k-1}}^{m}-v_{mb_{k-1}}^{m}\Delta t_{k}+\frac{1}{2}g^{m}\Delta t_{k}^{2}\right)-{\hat{\alpha }}_{b_{k}}^{b_{k-1}}\\ C_{m}^{b_{k-1}}\left(v_{mb_{k}}^{m}-v_{mb_{k-1}}^{m}+g^{m}\Delta t_{k}\right)-{\hat{\beta }}_{b_{k}}^{b_{k-1}}\\ {\left[\left({\left(q_{b_{k}}^{m}\right)}^{-1}⊗q_{b_{k-1}}^{m}\right)⊗{\hat{q}}_{b_{k}}^{b_{k-1}}\right]}_{xyz}\\ b_{a}\left(t_{k}\right)-b_{a}\left(t_{k-1}\right)\\ b_{g}\left(t_{k}\right)-b_{g}\left(t_{k-1}\right)\\ C_{m}^{b_{k-1}}\left(p_{mb_{k}}^{m}-p_{mb_{k-1}}^{m}\right)-{\hat{s}}_{b_{k}}^{b_{k-1}}\\ s_{odo}\left(t_{k}\right)-s_{odo}\left(t_{k-1}\right) \end{array}\right] \end{array}$$

where $\sigma_{z}^{2}$ is the variance covariance matrix of **z**, which can be obtained from Equation (22) [19,32]; ${\hat{\alpha }}_{b_{k}}^{b_{k-1}}$, ${\hat{\beta }}_{b_{k}}^{b_{k-1}}$, ${\hat{q}}_{b_{k}}^{b_{k-1}}$, and ${\hat{s}}_{b_{k}}^{b_{k-1}}$ are the corrected pre-integration results by the first-order approximation [30].

The amount of calculation for the back-end graph optimization gradually increased because the nodes and submaps increased significantly over time. In order to ensure that the number of parameters remained relatively stable, this paper used the sliding window method [33,34] to delete the oldest submap while adding a new submap. (See our previous work [19] for the detailed process.) At the same time, in order to output the navigation results in time, the position, velocity, and attitude of the latest node in the sliding window were used as the starting point. The navigation information that recursed to the latest IMU data time according to Equation (1) was used as the output. Meanwhile, the node’s IMU bias and odometry scale factor were fed back to the front-end pose estimation. The IMU and odometer data correction in the front-end pose estimation was performed with the latest feedback, as shown in Figure 2. The IMU bias and the odometer scale factor changed slowly, and the front- and back-end time differences were ignored.

## 3. Tests

**Figure 5:**
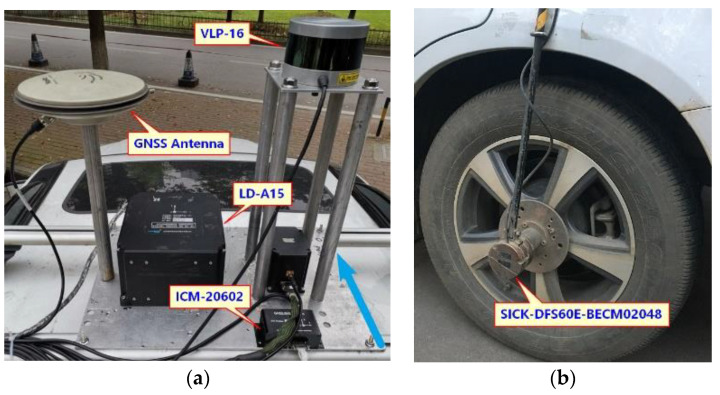
Testing platform. (**a**) GNSS, IMU and LiDAR (**b**) Odometer.

On 13 April and 18 July 2019, tests to evaluate the performance of the GNSS/IMU/ODO/LiDAR-SLAM integrated navigation system were carried out in the Fozuling and East Lake Tunnel, Wuhan. As shown in Figure 5, the test vehicle is equipped with a LiDAR (VLP-16), a low-cost MEMS-IMU (ICM-20602), and a navigation grade GNSS/INS integrated navigation system (LD-A15). Table 1 gives specifications for LD-A15 and ICM-20602. This paper used a 2048-resolution encoder (SICK-DFS60E-BECM02048) to collect odometer data. The sampling rate of the GNSS in the LD-A15 is 1 Hz, the sampling rate of the VLP-16 is 10 Hz (i.e., 600 RPM), and the sampling rates of the IMU in the LD-A15, the ICM-20602 and the odometer are all 200 Hz. The speed in the open-sky tests was about 10 m/s and the speed in the tunnel test was about 17 m/s.

**Figure 6:**
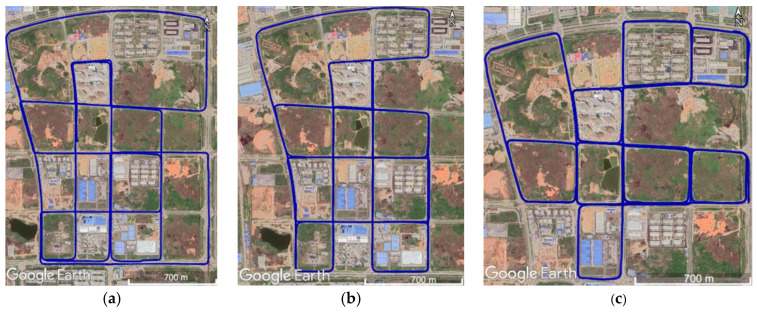
Trajectories of the open-sky tests. (**a**) Test #1, (**b**) Test #2, and (**c**) Test #3.

This paper adopted the statistic result of the maximum navigation error during GNSS signal outage to evaluate the accuracy of the proposed navigation system. The trajectories of the three tests conducted in the open-sky areas are shown in Figure 6. The reference truth value of the vehicle’s position and attitude was obtained from GNSS data and IMU data of the LD-A15 using the RTK (real time kinematic)/INS smoothing integration algorithm. Then, two minutes of GNSS outage was deliberately added into the GNSS RTK results every seven minutes to mimic satellite signals cut and resumed at the same time. Thereafter, the RTK results with GNSS outages, the ICM-20602 data, the VLP-16 data, and the odometer data were used for performance evaluation. In addition to the proposed integrated navigation method, for comparison, two other processing methods were conducted:

(1) GNSS/INS/ODO: the GNSS/INS integration method with the odometer and NHC constraint, to show the contribution of the LiDAR-SLAM.
(2) GNSS/IMU/LiDAR-SLAM: the proposed integrated method but without the odometer assistance, to show the contribution of adding the odometer into the pre-integration.

**Figure 7:**
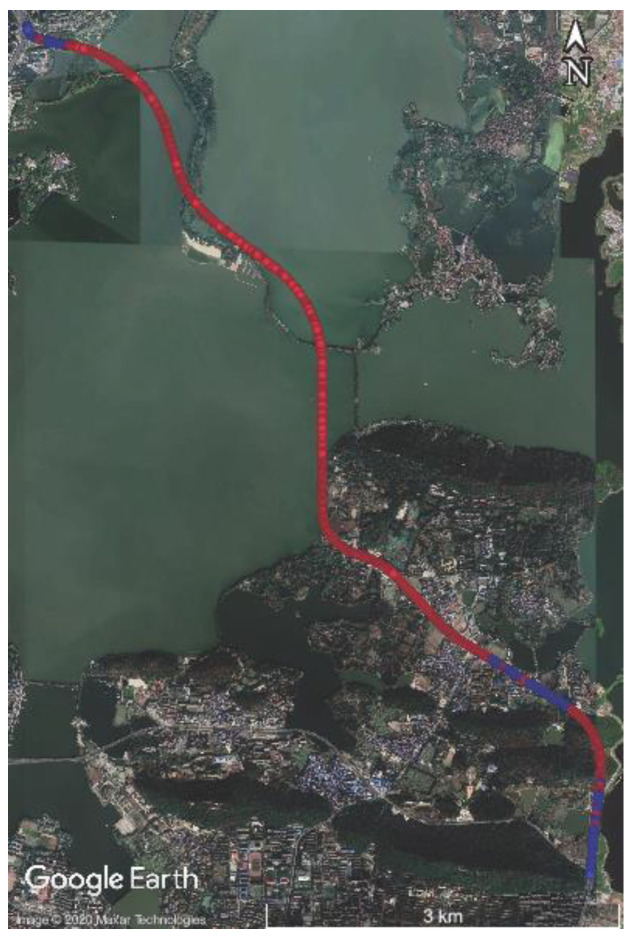
Trajectory of the East Lake Tunnel test.

The same processing was performed on the data in the East Lake Tunnel test. The total length of the East Lake Tunnel is 6795 m (the longest red part in Figure 7), in which the GNSS signal was interrupted in real case and the environmental features for LiDAR-SLAM were insufficient.

## 4. Results and Discussion

**Figure 8:**
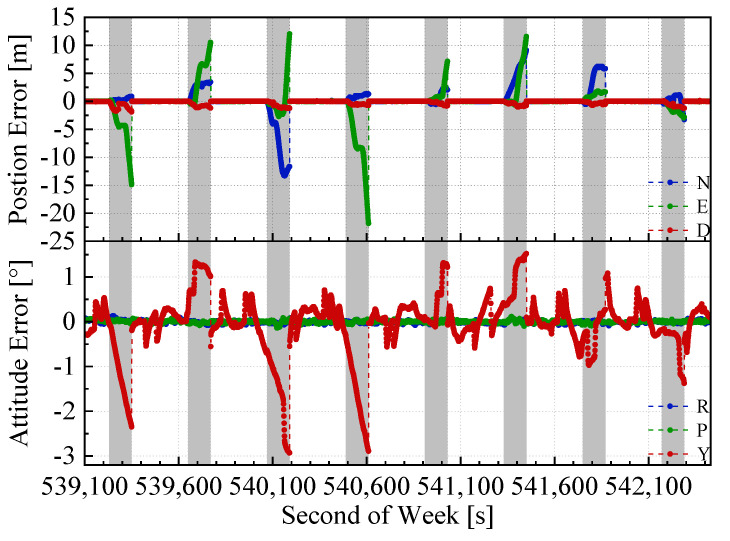
GNSS/INS/ODO navigation errors in test #2 (with 2 min GNSS outages).

**Figure 9:**
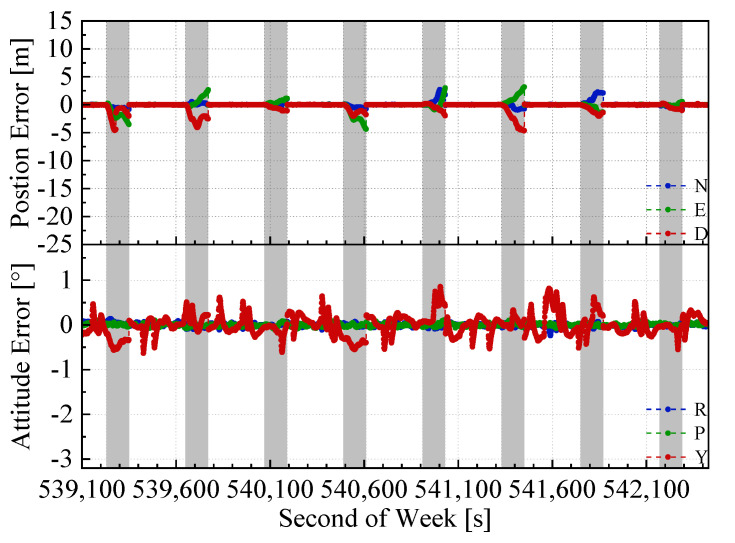
GNSS/IMU/LiDAR-SLAM navigation errors in the test #2 (with 2 min GNSS outages)

**Figure 10:**
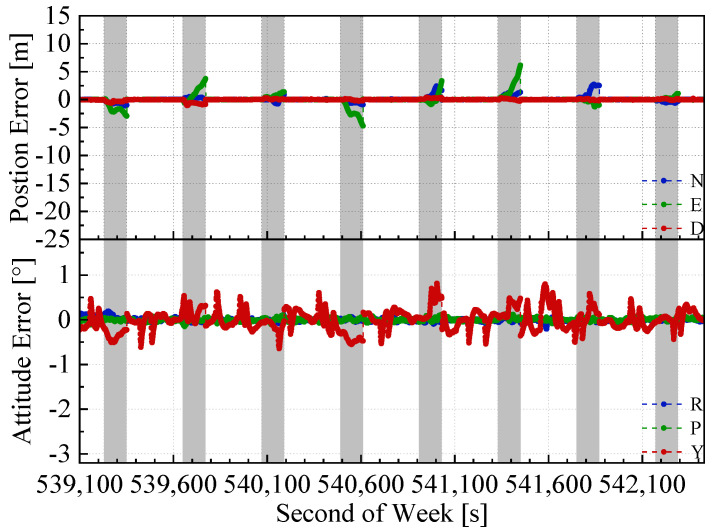
GNSS/IMU/ODO/LiDAR-SLAM navigation errors in test #2 (with 2 min GNSS outages).

The position and attitude errors of the proposed integrated navigation method and the two benchmarked methods with the mimic two minute GNSS outages are shown in Figure 8, Figure 9 and Figure 10. Here, we used the open sky test #2 as an example. The grey span in the figures marks the mimic GNSS outages periods. From the navigation error graphs, the following can be observed:

(1) The GNSS/INS/ODO integrated navigation system had the largest navigation errors, especially for heading errors. During the 1st, 3rd, and 4th outages in the figures, when the vehicle moved with uniform speed along a straight line, it can be seen that despite with the NHC assistance, the heading error of the GNSS/INS/ODO integrated navigation system was still much larger than the other two methods with LiDAR-SLAM assistance. The LiDAR-SLAM proposed in the paper had a slower drift rate than the INS/ODO dead reckoning trajectory and also maintained the heading estimation effectually during GNSS outages.
(2) In the open-sky areas, the surrounding buildings and trees were rich in features and LiDAR-SLAM worked well to maintain the horizontal positioning and attitude. Comparing Figure 9 and Figure 10 shows the contribution of the odometer and NHC. With the presence of good LiDAR-SLAM, the odometer had little effect on attitude and horizontal positioning errors, but the NHC helped reduce height errors significantly.

There were 21 mimic GNSS outages in the three open-sky tests, whose average mileage was 827 m. According to the error statistics during the outages, as shown in Table 2, the error level of the GNSS/IMU/ODO/LiDAR-SLAM integrated navigation system can be observed:

(1) Compared with the GNSS/INS/ODO integrated navigation system, the position error RMS was reduced by 62.8%, 72.3%, and 52.1%; the heading error RMS was reduced by 62.1%; and the roll and pitch errors were equivalent.
(2) Compared to the GNSS/IMU/LiDAR-SLAM integrated navigation system, the position errors RMS in the north and east directions were equivalent (1.9 m and 2.5 m, respectively). The vertical position error was reduced by 72.3% and the RMS of roll, pitch, and heading errors were equivalent (0.1°, 0.1° and 0.6°, respectively).

**Figure 11:**
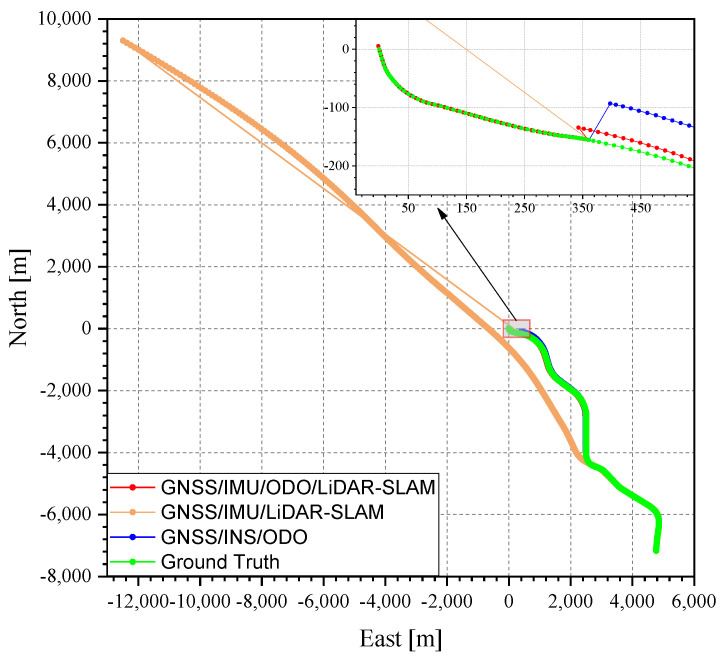
Trajectories in the East Lake Tunnel test.

**Figure 12:**
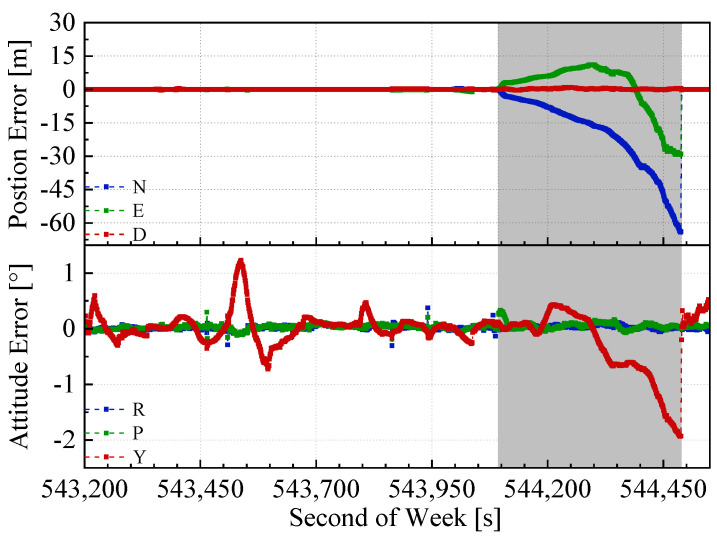
GNSS/INS/ODO navigation errors in the East Lake Tunnel test.

**Figure 13:**
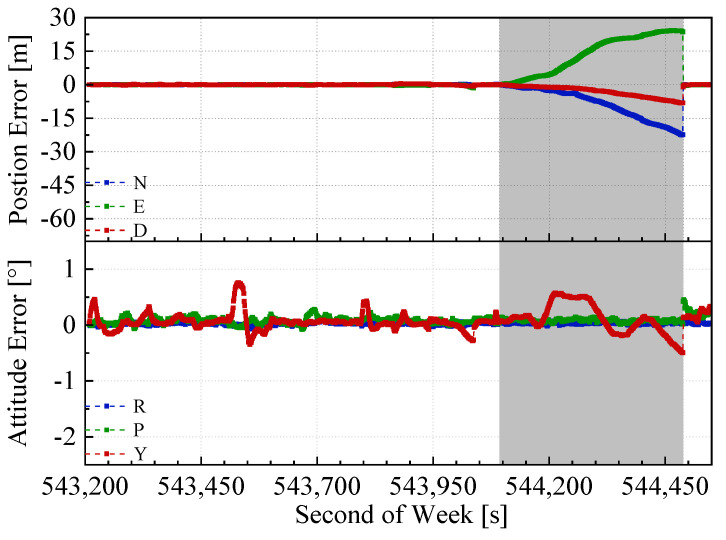
GNSS/IMU/ODO/LiDAR-SLAM navigation errors in the East Lake Tunnel test.

The mimic GNSS outages tests could not replace the real GNSS outages case (i.e., tunnel) because of the difference, such as (1) GNSS fading before real outages and (2) the LiDAR-SLAM degradation in the tunnel because of the lack of environmental features. Therefore, the same test and comparison was performed in the Wuhan East Lake Tunnel. The trajectories in the test are shown in Figure 11. As can be seen from Figure 11, the GNSS/IMU/LiDAR-SLAM integrated navigation system drifted far away from the true trajectory because the LiDAR-SLAM did not work normally in the tunnel with insufficient environmental features, and there was no GNSS signals. The MEMS-IMU was not able to maintain the trajectory alone for such a long time (about 400s). Therefore, the follow-up navigation error analysis only contained the remaining two methods, as shown in Figure 12 and Figure 13. The grey span in the figures marks the tunnel part.

It can be seen from Table 3 that the north, east, and height errors in the end of the tunnel of the GNSS/INS/ODO integrated navigation system were 64.1, 28.9, and 0.4 m, while the north, east, and height errors of the GNSS/IMU/ODO/LiDAR-SLAM integrated navigation system were 22.3, 23.6, and 8.1 m, respectively. The heading error of the GNSS/IMU/ODO/LiDAR-SLAM navigation error was 0.5°, which was much less than that of the GNSS/INS/ODO integrated navigation system (about 2°). Such heading difference also met the results of the open-sky tests with mimic GNSS outages. Although the LiDAR-SLAM did not work well in the tunnel with insufficient features, the inner wall of the tunnel effectually constrained the position drift along the lateral direction of the tunnel and maintained the heading estimation. The insufficient feature of the tunnel mainly referred to the missing constraint along the forward direction and caused forward position drifting (as shown in Figure 11). However, such an issue was solved by introducing the odometer aiding (through the IMU/ODO pre-integration), which effectually constrained forward drifting. Therefore, the IMU/ODO pre-integration proposed in this paper provided an essential aiding to the LiDAR-SLAM in tunnel scenarios.

## 5. Conclusions

In order to solve navigation in areas with poor GNSS signals and insufficient environmental features, this paper proposed a GNSS/IMU/ODO/LiDAR-SLAM integrated navigation system. IMU and odometer data were used to perform pose recursion. The relative pose from LiDAR-SLAM, GNSS position, and IMU/ODO pre-integration results were fused through graph optimization. In addition, in order to prevent the calculation amount of graph optimization from increasing over time, the sliding window was applied to keep the number of optimized variables relatively stable. The GNSS outage tests showed that, compared with the GNSS/INS/ODO integrated navigation system, the assistance of LiDAR-SLAM effectually reduced position and heading errors. The RMS of the position errors in the north, east, and height were reduced by 62.8%, 72.3%, and 52.1%. The RMS of the heading error was reduced by 62.1%. The RMS of the roll and pitch error were equivalent. Compared to the GNSS/IMU/LiDAR-SLAM integrated navigation system, the assistance of the odometer reduced the height error by 72.3%. The horizontal position and attitude error were equivalent. The auxiliary effects of the LiDAR-SLAM and the odometer were not essentially different because both reckoned with divergence. However, in environments where LiDAR-SLAM did not work effectually (e.g., inside tunnel), the assistance of the odometer was particularly necessary and important, while the LiDAR-SLAM had the advantage of mitigating the lateral drift and heading drift. The tunnel test verified the contributions of the LiDAR-SLAM and the odometer in the proposed GNSS/IMU/ODO/LiDAR-SLAM integrated navigation system. For future work, the dynamic object recognition and tracking module should be used to eliminate the interference of dynamic objects in the environment to the SLAM system, and thereby further improve the robustness of the navigation system.

## Figures and Tables

**Table 1:** Specifications of the LD-A15 and ICM-20602.

IMU	Accelerometer		Gyroscope	
	Bias Instability [mGal]	Random Walk Noise $\left[m/s/\sqrt{h}\right]$	Bias Instability [°/h]	Random Walk Noise $\left[{}^{\circ}/\sqrt{h}\right]$
LD-A15	15	0.03	0.027	0.003
ICM-20602	250	0.24	50	0.24

**Table 2:** Statistical analysis of the open-sky tests with 2 min GNSS outages.

		Position Error [m]			Attitude Error [°]		
		N	E	D	R	P	Y
GNSS/INS/ODO	RMS	5.2	9.1	1.1	0.11	0.10	1.59
	MAX	13.3	21.8	1.9	0.22	0.20	2.93
GNSS/IMU/LiDAR-SLAM	RMS	1.9	2.5	1.9	0.13	0.11	0.60
	MAX	3.6	4.4	4.6	0.33	0.19	0.99
GNSS/IMU/ODO/LiDAR-SLAM	RMS	1.9	2.5	0.5	0.11	0.11	0.60
	MAX	3.6	6.1	1.4	0.19	0.18	1.15

**Table 3:** The navigation errors in the end of the tunnel.

	Position Error [m]			Attitude Error [°]		
	N	E	D	R	P	Y
GNSS/INS/ODO	−63.8	−28.9	0.4	0.01	0.02	−1.93
GNSS/IMU/LiDAR-SLAM	9520.6	12993.5	1428.9	−0.80	4.86	3.50
GNSS/IMU/ODO/LiDAR-SLAM	−22.4	23.6	−8.1	0.02	0.01	−0.49

## References

[1] Shin E.-H. (2001). Accuarcy Improvement of Low Cost INS/GPS for Land Applications. Master’s Thesis.

[2] Cadena C., Carlone L., Carrillo H., Latif Y., Scaramuzza D., Neira J., Reid I., Leonard J.J. (2016). Past, present, and future of simultaneous localization and mapping: Toward the robust-perception age. IEEE Trans. Robot..

[3] Martínez J.L., González J., Morales J., Mandow A., García-Cerezo A.J. (2006). Mobile robot motion estimation by 2D scan matching with genetic and iterative closest point algorithms. J. Field Robot..

[4] Zhang J., Singh S. LOAM: Lidar Odometry and Mapping in Real-time. Proceedings of the 2014 Robotics: Science and Systems.

[5] Shan T., Englot B. Lego-loam: Lightweight and ground-optimized lidar odometry and mapping on variable terrain. Proceedings of the 2018 IEEE/RSJ International Conference on Intelligent Robots and Systems (IROS).

[6] Liu X., Zhang L., Qin S., Tian D., Ouyang S., Chen C. (2019). Optimized LOAM Using Ground Plane Constraints and SegMatch-Based Loop Detection. Sensors.

[7] Censi A. An ICP variant using a point-to-line metric. Proceedings of the 2008 IEEE International Conference on Robotics and Automation.

[8] Segal A., Haehnel D., Thrun S. Generalized-icp. Proceedings of the 2009 Robotics: Science and Systems.

[9] Burguera A., González Y., Oliver G. (2009). On the use of likelihood fields to perform sonar scan matching localization. Auton. Robot..

[10] Hess W., Kohler D., Rapp H., Andor D. Real-time loop closure in 2D LIDAR SLAM. Proceedings of the 2016 IEEE International Conference on Robotics and Automation (ICRA).

[11] Qian C., Liu H., Tang J., Chen Y., Kaartinen H., Kukko A., Zhu L., Liang X., Chen L., Hyyppä J. (2017). An integrated GNSS/INS/LiDAR-SLAM positioning method for highly accurate forest stem mapping. Remote Sens..

[12] Gao Y., Liu S., Atia M.M., Noureldin A. (2015). INS/GPS/LiDAR integrated navigation system for urban and indoor environments using hybrid scan matching algorithm. Sensors.

[13] Chiang K.-W., Tsai G.-J., Li Y.-H., Li Y., El-Sheimy N. (2020). Navigation Engine Design for Automated Driving Using INS/GNSS/3D LiDAR-SLAM and Integrity Assessment. Remote Sens..

[14] Shamsudin A.U., Ohno K., Hamada R., Kojima S., Westfechtel T., Suzuki T., Okada Y., Tadokoro S., Fujita J., Amano H. (2018). Consistent map building in petrochemical complexes for firefighter robots using SLAM based on GPS and LIDAR. Robomech J..

[15] Kukko A., Kaijaluoto R., Kaartinen H., Lehtola V.V., Jaakkola A., Hyyppä J. (2017). Graph SLAM correction for single scanner MLS forest data under boreal forest canopy. ISPRS J. Photogramm. Remote Sens..

[16] Cartographer. https://github.com/cartographer-project/cartographer.

[17] Chiang K., Tsai G., Chu H., Elsheimy N. (2020). Performance Enhancement of INS/GNSS/Refreshed-SLAM Integration for Acceptable Lane-Level Navigation Accuracy. IEEE Trans. Veh. Technol..

[18] Durrant-Whyte H., Bailey T. (2006). Simultaneous localization and mapping: Part I. IEEE Robot. Autom. Mag..

[19] Chang L., Niu X., Liu T., Tang J., Qian C. (2019). GNSS/INS/LiDAR-SLAM Integrated Navigation System Based on Graph Optimization. Remote Sens..

[20] Shin E.-H., Estimation Techniques for Low-Cost Inertial Navigation (2005). UCGE Report. https://www.ucalgary.ca/engo_webdocs/NES/05.20219.EHShin.pdf.

[21] Sukkarieh S. (2000). Low Cost, High Integrity, Aided Inertial Navigation Systems for Autonomous Land Vehicles. Ph.D. Thesis.

[22] Dissanayake G., Sukkarieh S., Nebot E., Durrant-Whyte H. (2001). The aiding of a low-cost strapdown inertial measurement unit using vehicle model constraints for land vehicle applications. IEEE Trans. Robot. Autom..

[23] Wu Y., Wu M., Hu X., Hu D. Self-calibration for land navigation using inertial sensors and odometer: Observability analysis. Proceedings of the AIAA Guidance Navigation, and Control Conference.

[24] Zhang S., Guo Y., Zhu Q., Liu Z. Lidar-IMU and Wheel Odometer Based Autonomous Vehicle Localization System. Proceedings of the Chinese Control and Decision Conference.

[25] Meng X., Wang H., Liu B. (2017). A Robust Vehicle Localization Approach Based on GNSS/IMU/DMI/LiDAR Sensor Fusion for Autonomous Vehicles. Sensors.

[26] Chen Q., Zhang Q., Niu X. (2020). Estimate the Pitch and Heading Mounting Angles of the IMU for Land Vehicular GNSS/INS Integrated System. IEEE Trans. Intell. Transp. Syst..

[27] Agarwal S., Mierle K. Ceres-Solver. http://ceres-solver.org.

[28] Xiangyuan K., Jiming G., Zongquan L. (2010). Foundation of Geodesy.

[29] Le Gentil C., Vidal-Calleja T., Huang S. 3d lidar-imu calibration based on upsampled preintegrated measurements for motion distortion correction. Proceedings of the 2018 IEEE International Conference on Robotics and Automation (ICRA).

[30] Qin T., Li P., Shen S. (2018). VINS-Mono: A Robust and Versatile Monocular Visual-Inertial State Estimator. IEEE Trans. Robot..

[31] Jun W., Gongmin Y. (2016). Strapdown Inertial Navigation Algorithm and Integrated Navigation Principles.

[32] Yongyuan Q. (2000). Kalman Filter and Integrated Navigation Principle.

[33] Sibley G., Matthies L., Sukhatme G.S. (2010). Sliding window filter with application to planetary landing. J. Field Robot..

[34] Eckenhoff K., Paull L., Huang G. Decoupled, consistent node removal and edge sparsification for graph-based SLAM. Proceedings of the 2016 IEEE/RSJ International Conference on Intelligent Robots and Systems (IROS).

